# Mitochondrial metabolism and bioenergetic function in an anoxic isolated adult mouse cardiomyocyte model of *in vivo* cardiac ischemia-reperfusion injury

**DOI:** 10.1016/j.redox.2022.102368

**Published:** 2022-06-17

**Authors:** Anja V. Gruszczyk, Alva M. Casey, Andrew M. James, Hiran A. Prag, Nils Burger, Georgina R. Bates, Andrew R. Hall, Fay M. Allen, Thomas Krieg, Kourosh Saeb-Parsy, Michael P. Murphy

**Affiliations:** a MRC Mitochondrial Biology Unit, Biomedical Campus, University of Cambridge, Cambridge, CB2 0XY, UK; b Department of Surgery and Cambridge NIHR Biomedical Research Centre, Biomedical Campus, University of Cambridge, Cambridge, CB2 2QQ, UK; c NIHR Biomedical Research Centre and NIHR Blood and Transplant Research Unit in Organ Donation and Transplantation, Cambridge Biomedical Campus, Cambridge, UK; d Department of Medicine, University of Cambridge, Cambridge, CB2 0QQ, UK

**Keywords:** Mitochondria, Ischemia-reperfusion injury, Metabolism, Cardiomyocytes, Hydrogen peroxide

## Abstract

Cell models of cardiac ischemia-reperfusion (IR) injury are essential to facilitate understanding, but current monolayer cell models poorly replicate the *in vivo* IR injury that occurs within a three-dimensional tissue. Here we show that this is for two reasons: the residual oxygen present in many cellular hypoxia models sustains mitochondrial oxidative phosphorylation; and the loss of lactate from cells into the incubation medium during ischemia enables cells to sustain glycolysis. To overcome these limitations, we incubated isolated adult mouse cardiomyocytes anoxically while inhibiting lactate efflux. These interventions recapitulated key markers of *in vivo* ischemia, notably the accumulation of succinate and the loss of adenine nucleotides. Upon reoxygenation after anoxia the succinate that had accumulated during anoxia was rapidly oxidized in association with extensive mitochondrial superoxide/hydrogen peroxide production and cell injury, mimicking reperfusion injury. This cell model will enable key aspects of cardiac IR injury to be assessed *in vitro*.

## 1 Introduction

*In vitro* cardiomyocyte models of ischemia-reperfusion (IR) injury deepen our understanding by enabling experiments that are not possible *in vivo* or in the isolated, perfused heart. However, investigations of IR injury using cardiomyocytes *in vitro* are limited because they only moderately replicate key aspects of ischemia such as the profound changes in succinate, lactate and adenine nucleotides that are seen during ischemia *in vivo* [1,2] and in isolated hearts [[3], [4], [5]]. To address this, we set out to develop a model of IR injury in isolated adult cardiomyocytes that more closely mimics the distinctive metabolic signature that occurs during cardiac ischemia *in vivo*.

The lack of blood supply to the heart during ischemia, coupled with the very low apparent K_m_ of cytochrome oxidase (∼1 μM [6,7]) for O_2_ means that mitochondria within ischemic tissues keep the O_2_ concentration at negligible levels [8]. In contrast, the O_2_ levels used in most hypoxic cell incubators are 1–0.1%, which corresponds to an [O_2_] of 10–1 μM [9]. While these O_2_ levels can activate the HIF-1α pathway due to the high K_m_ of prolyl hydroxylase 2 (PHD2) for O_2_ (∼230–250 μM) [10]), they provide sufficient O_2_ to maintain mitochondrial respiration. Furthermore, *in vitro* cells are monolayers exposed to an O_2_ reservoir in the medium far larger than they can deplete, so its diffusion into cells will sustain mitochondrial oxygen-consumption indefinitely. Thus, in stark contrast to ischemia *in vivo,* mitochondria in hypoxic cell models are still respiring and will continue to do so for prolonged periods.

A further difference between ischemia in tissues and cells in culture is that the lack of blood flow *in vivo*, coupled with the low volume of extracellular fluid, means that during tissue ischemia glucose supply is constrained. Consequently, lactate from anaerobic glycolysis will accumulate in tissues, both within cells and in the extracellular fluid. This will lead to a build-up of NADH within the cells of the tissue, slowing of glycolysis at GAPDH and decreased glycolytic ATP production [4]. In contrast, the large volume of incubation medium in which cardiomyocytes are cultured acts as a sink for both lactate and protons, both of which are removed from cells in symport via monocarboxylate transporters (MCT) [11]. This continual lactate efflux allows the cell to maintain a lower NADH/NAD^+^ ratio and thus sustain ATP synthesis by glycolysis. Some cardiomyocyte models circumvent this by using a glucose-free lactate-based buffer. However, these conditions are different from those found during ischemia *in vivo* where tissues utilize glycogen stores. We reasoned that preventing lactate efflux from cells *in vitro* through pharmacological inhibition of the MCT carriers would better mimic what is occurring *in vivo* within tissues with low amounts of extracellular space that rapidly saturate with accumulated metabolites. This intervention should impede glycolysis at GAPDH by increasing the intracellular NADH/NAD^+^ ratio, better mimicking *in vivo* ischemia than through the removal of glucose. It will also allow for an increase in intracellular lactate and a decrease in intracellular pH. Finally, during the isolation of adult cardiomyocytes their glycogen stores will likely become depleted [12] therefore, glucose should be supplied in the medium for anoxic cell incubations.

**Fig. 1:**
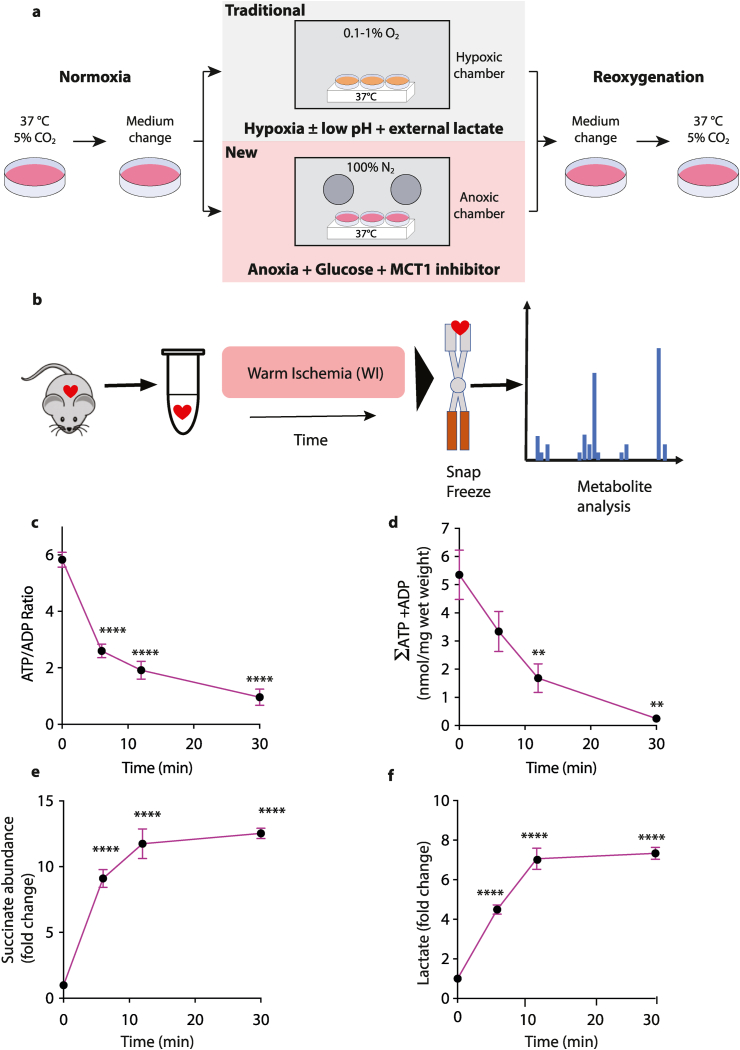
(a) Primary murine adult cardiomyocytes were isolated from freshly retrieved hearts and cultured overnight at 37 °C, 5% CO_2_. Then cells were washed and transferred into glucose- or low pH/lactate-based experimental buffer, degassed and moved either into hypoxia or into the anoxic chamber. The cells were either maintained and lysed within the chambers, or reoxygenated by removal from the anoxic chamber and given fresh oxygenated incubation medium. (b–f) Hallmarks of ischemia and reperfusion within the mouse heart. (b) Schematic illustrating the experimental design to induce cardiac ischemia in mouse hearts. The whole heart (∼150–180 mg), was either immediately clamp frozen at liquid nitrogen temperature to generate a baseline sample under normoxic conditions, or incubated to induce warm ischemia (WI, 37 °C) for the indicated times prior to freeze clamping. Tissues were then extracted and metabolites assayed. ATP and ADP were determined and the ATP/ADP ratio (c) and the sum of ATP and ADP (nmol/mg wet weight) (d) calculated. n = 5–11. (e) Heart succinate levels during WI. Data are fold change relative to normoxia (n = 6). (f) Heart lactate levels during WI. Data are fold-change relative to normoxia, n = 4. Statistical analysis: one-way ANOVA with Tukey's *post hoc* test, **P < 0.01, ****<0.0001. Data are reproduced from Ref. [4].

Therefore, we hypothesized that incubating cardiomyocytes anoxically, while inhibiting lactate efflux and supplying external glucose, would replicate key aspects of *in vivo* heart ischemia (Fig. 1a). Here we establish this model and show that it replicates key metabolic signatures of cardiac ischemia *in vivo* while hypoxic incubations do not. Reoxygenation of these anoxic cells causes damage similar to reperfusion injury and enables changes in mitochondrial function following IR injury to be investigated.

## 2 Results

### 2.1 In vitro model of ischemia in adult cardiomyocytes

Ischemia occurs when blood flow to a tissue is blocked. While this prevents the delivery of oxygen, it also prevents the supply and removal of other metabolites. Ischemia in the heart is characterised by several key metabolic changes in lactate, succinate and adenine nucleotides. These variables are robust markers of ischemia in the intact organ because they alter significantly, and to similar extents, within ischemic mouse, pig and human hearts [[1], [2], [3], [4], [5]]. For example, our previously published ischemic data (shown in purple) [4] show that within ischemic mouse hearts (Fig. 1b) there is a decrease in the ATP/ADP ratio (Fig. 1c), a loss of adenine nucleotides (Fig. 1d), and accumulation of both succinate and lactate (Fig. 1e and f). For an isolated cardiomyocyte system to recapitulate ischemia in an intact heart it will at the very least require similar changes in these metabolites in cardiomyocytes. To assess whether *in vitro* cardiomyocyte incubation conditions mimicked those in the ischemic heart, we measured changes in lactate, succinate, ATP/ADP ratio and the size of the ATP and ADP pools. We hypothesized that incubating adult cardiomyocytes under anoxia (0.1–10 ppm O_2_; 0.1–10 nM O_2_) rather than hypoxia (∼0.1%/1000 ppm O_2_; ∼1 μM O_2_), while inhibiting lactate efflux through MCT carriers and supplying external glucose would lead to similar changes in these markers and thus better mimic *in vivo* heart ischemia (Fig. 1a).

**Fig. 2:**
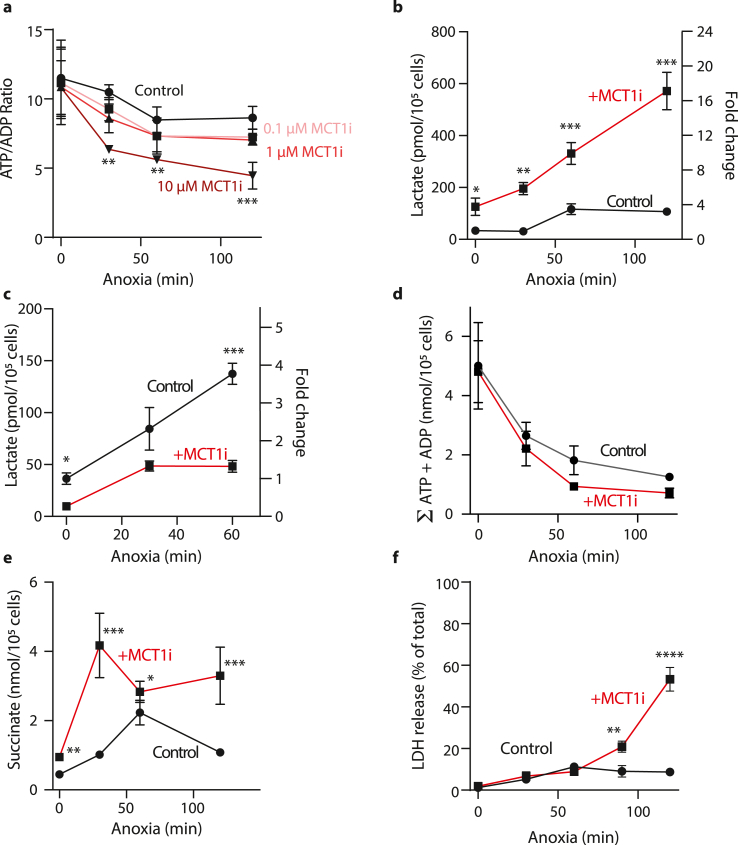
Cardiomyocytes were incubated for different time periods of anoxia or 120 min normoxia at 37 °C with various concentration of AR-C141990. Control buffer with 0 μM MCT1 inhibitor is shown in black, MCT1 inhibitor-containing buffer in red. The supernatant and cell pellets were then isolated and analyzed. (a) Cell ATP/ADP ratio (n = 3–5 biological replicates). Statistical significance was assessed by one-way ANOVA with multiple comparisons and Tukey's *post hoc* test, **P < 0.005, ***P = 0.002. (b) Cell lactate concentration (pmol/10^5^ cells) on the left y-axis and lactate fold changes to control on the right y-axis (n = 3–5 biological replicates, *P < 0.03, **P < 0.01, ***P < 0.0005, one-way ANOVA with multiple comparisons and Tukey's *post hoc* test. (c) Cell supernatant lactate: lactate concentration on the left y-axis and lactate fold changes to control on the right y-axis. n = 3–5 biological replicates, *P < 0.05, ***P < 0.0005 one-way ANOVA with multiple comparisons and Tukey's *post hoc* test. (d) Cells were incubated with 0 or 10 μM AR-C141990 and the sum of the cell ATP and ADP contents determined. (e) Cell succinate levels (nmol/10^5^ cells); n = 5–11 biological replicates, *P < 0.05, **P < 0.0005, ***P = 0.0002 one-way ANOVA with multiple comparisons and Tukey's *post hoc* test. (f) Cell death was evaluated by measuring the amount of LDH released into the cellular supernatant. Results are presented relative to a positive control of cardiomyocytes lysed in 1% Triton. n = 5–7 biological replicates. (For interpretation of the references to color in this figure legend, the reader is referred to the Web version of this article.)

To assess whether anoxic incubation of cardiomyocytes alone was sufficient to mimic *in vivo* ischemia, we measured changes in the ATP/ADP ratio (Fig. 2a), lactate levels in the cells (Fig. 2b), as well as lactate levels in the extracellular medium (Fig. 2c), the sum of ATP and ADP (Fig. 2d) and succinate (Fig. 2e) levels in the cells over 2 h of anoxia. While there were changes in all these variables, their extents were generally far less than in the ischemic heart (Fig. 1; Table 1). Therefore, we next inhibited lactate transport through MCT pharmacologically. The dominant MCT in cardiomyocytes is MCT1 [13], so we used the selective MCT1 inhibitor AR-C141990 [14] in conjunction with anoxia (data shown in red) and found that 10 μM AR-C141990 lowered the ATP/ADP ratio (Fig. 2a). Use of another MCT inhibitor which affects both MCT1 and MCT2 (AR-C155858) gave very similar results (data not shown). An AR-C141990 concentration of 10 μM also increased cell lactate levels (Fig. 2b), and decreased its efflux into the extracellular medium (Fig. 2c), consistent with inhibition of MCT1. The combination of anoxia and MCT1 inhibition also decreased the sum of ATP and ADP (Fig. 2d), and increased cell succinate (Fig. 2e). Comparing these changes with those in the ischemic mouse heart (Fig. 1) shows that incubating cardiomyocytes anoxically in conjunction with MCT1 inhibition while supplying glucose better reflects key metabolic changes that occur in the ischemic heart. Furthermore, under these conditions there was relatively little cell death at the 60 min time point (Fig. 2f), facilitating the use of this model to assess cell death upon reoxygenation as a model of IR injury.

**Table 1:** Comparison of fold change in adenosine nucleotides and metabolites between normoxic heart tissue and heart tissue after 30 min ischemia; and between normoxic cardiomyocytes and cardiomyocytes incubated under either: anoxia with MCT1 inhibition; hypoxia; or under low pH/high lactate/anoxia conditions for 30 min.

Fold-change vs Normoxia		Cardiomyocytes		
30 min	Ischemia	Anoxia	Hypoxia	Low pH/lactate Buffer
**ATP/ADP**	0.165	0.395	0.711	0.422
**Sum of nucleotides**	0.046	0.277	0.430	0.328
**Succinate**	12.533	8.350	1.589	6.071
**Lactate**	7.341	9.736	0.850	–

**Fig. 3:**
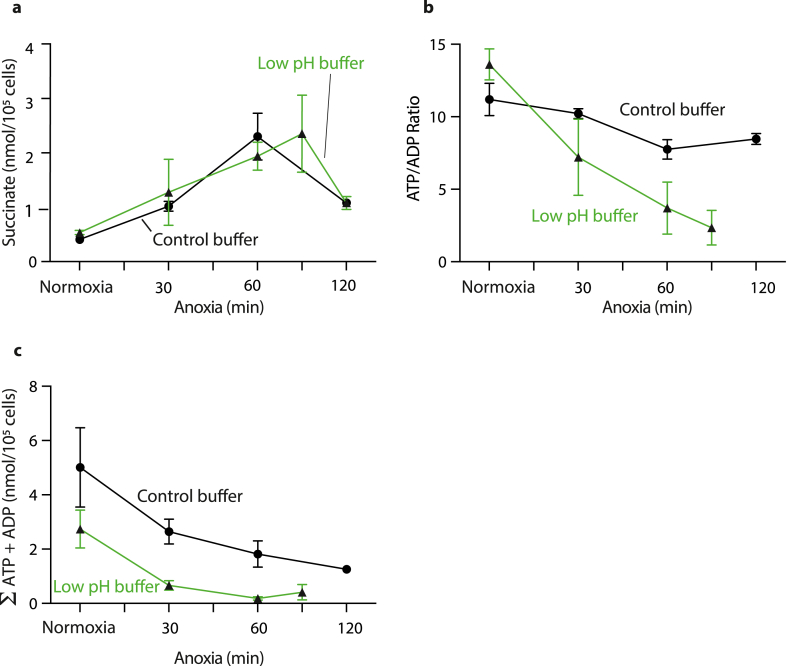
Cardiomyocytes were incubated for different times of anoxia, or for 120 min under normoxia, at 37 °C in the low pH (6.2)/high lactate buffer (green) under anoxia and compared with control buffer at pH 7.4 without MCT1 inhibition (black) (data from Fig. 2 a, d, e). (a) Cellular succinate (nmol/10^5^ cells). Data n = 3–5 biological replicates. (b) ATP/ADP ratio and (c) Sum of adenosine nucleotide concentrations (nmol/10^5^ cells). n = 2–5 biological replicates, data are mean ± S.D. or range for n = 2. (For interpretation of the references to color in this figure legend, the reader is referred to the Web version of this article.)

Within ischemic tissues there is a gradual decrease in pH due to anaerobic glycolysis [15,16]. These conditions have been replicated previously by using a high lactate buffer at pH 6.2 from the onset of hypoxia [17]. We next assessed how incubating cardiomyocytes during anoxia under these conditions affected their metabolism (Fig. 3), with data shown in green. The increase in succinate during anoxia at low pH/high lactate was comparable to that at pH 7.4 in the absence of MCT1 inhibition (Fig. 3a), but was lower than that at pH 7.4 with MCT1 inhibition (Fig. 2e). There was a greater decrease in the ATP/ADP in cells in low pH buffer than at pH 7.4 in the absence of MCT1 inhibition (Fig. 3b) which was also greater than that at pH 7.4 + MCT1 inhibition (Fig. 2a). There was a greater decrease in the total nucleotide concentrations in lactate buffer than at pH 7.4 in the absence of MCT inhibition (Fig. 3c) that was also greater than that at pH 7.4 + MCT1 inhibition (Fig. 2d). Together, these findings suggest that the gradual metabolic changes in our anoxic system with MCT1 inhibition, particularly that in succinate, better mimic *in vivo* ischemia, compared to using a step change to a low pH/high lactate buffer from the outset of an anoxic incubation.

**Fig. 4:**
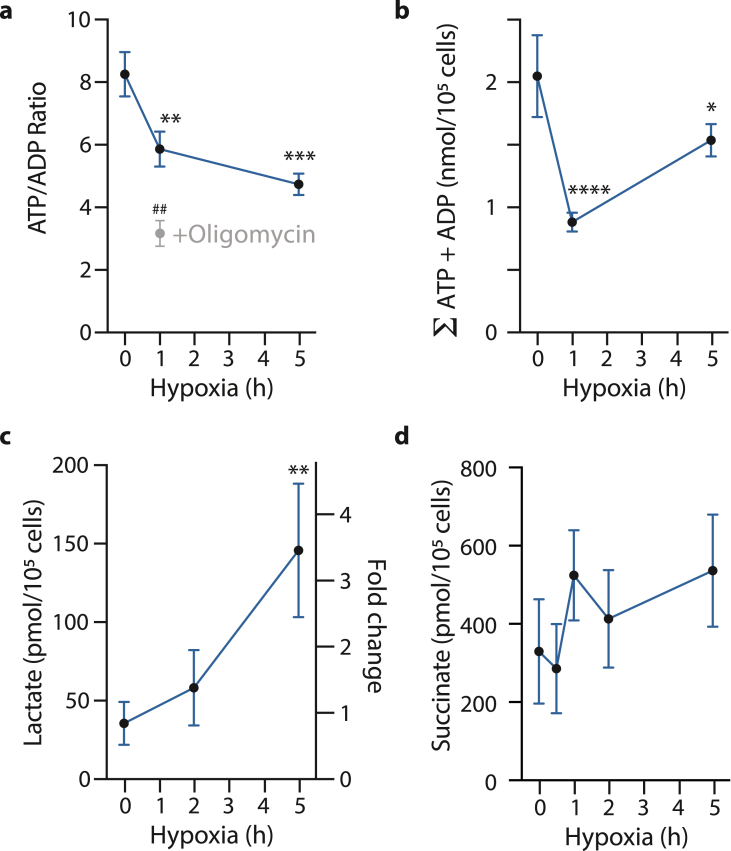
Changes in metabolic ischemic markers in primary murine cardiomyocytes were evaluated utilizing a hypoxic system at 0.1% O_2_, 5% CO_2_ at 37 °C in a CLARIOStar plate reader. The cells were cultured on laminated 6-well plates 24 h before hypoxia was induced for 1–5 h in Tyrode's buffer +10 μM AR-C141990, and, if indicated, 2 μM oligomycin was added 15 min before the end of hypoxia. Cells were lysed immediately upon retrieval from the plate reader and ATP/ADP ratios (a), adenosine nucleotide pools as the sum of ATP and ADP (nmol/10^5^ cells), (b), l-Lactate within cells in pmol/10^5^ cells on the left y-axis or as fold change on the right one (c), as well as intracellular succinate (nmol/10^5^ cells) (d) determined. n = 3–4 biological replicates. Statistical significance was assessed by one-way ANOVA with multiple comparisons and Tukey's *post hoc* test. *P < 0.05, **P < 0.01, ***P < 0.001, ****P < 0.0001, ^##^P < 0.01 + oligomycin vs control after 1 h hypoxia.

**Fig. 5:**
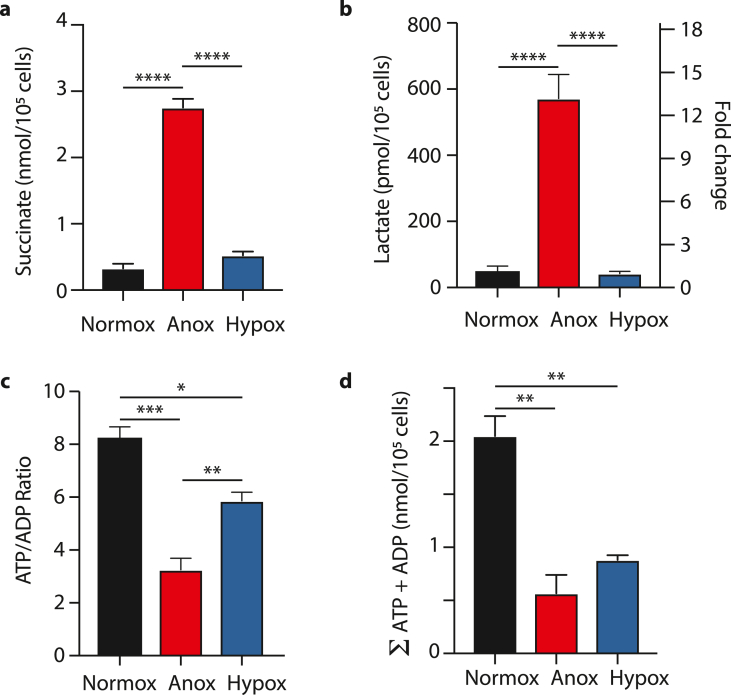
Comparison of cellular changes during normoxia, anoxia and hypoxia. Cardiomyocytes (in buffer + 10 μM AR-C141990) were either maintained at 37 °C, 5% CO_2_ in a cell incubator (normox), in an anoxic chamber at 37 °C (anox) or at 0.1% O_2_ at 37 °C (hypox) for 1 h (a,c,d) or 2 h (b). Succinate in nmol/10^5^ cells (a) and l-Lactate in pmol/10^5^ cells on the left or as fold change on the right y-axis (b). ATP/ADP ratios (c), and sum of adenosine nucleotides (ATP + ADP) in nmol/10^5^ cells (d) n = 3–4 biological replicates, statistical test: one-way ANOVA with multiple comparisons and Tukey's *post hoc* test, *P < 0.05, **P < 0.008, ***P < 0.003, ****P < 0.0001.

We next compared the effects of anoxia on cardiomyocytes with those of hypoxia at the lowest level (∼0.1% O_2_) typically used in cell models of ischemia. To do this we incubated cardiomyocytes with the MCT1 inhibitor in a hypoxic (0.1% O_2_) cell incubator (Fig. 4), with data shown in blue. Incubation at 0.1% O_2_ for 2–5 h led to only minor changes in the ATP/ADP ratio (Fig. 4a), adenine nucleotide levels (Fig. 4b), lactate (Fig. 4c) and succinate (Fig. 4d), compared to anoxic incubations. Furthermore, the ATP/ADP ratio was decreased by oligomycin (Fig. 4a), confirming that even under hypoxic conditions oxidative phosphorylation was still operating. The dramatic differences between the effects of anoxic and hypoxic incubations in the presence of MCT1i are shown for succinate (Fig. 5a), lactate (Fig. 5b) and ATP/ADP (Fig. 5c). Interestingly, there was no major change in the sum of adenine nucleotides between anoxia and hypoxia (Fig. 5d). We conclude that incubation of isolated adult cardiomyocytes under anoxic, but not hypoxic, conditions in conjunction with a glucose supply and inhibition of lactate transport by MCT1 better reproduces the key metabolic changes that occur in the ischemic heart (Table 1).

### 2.2 Mimicking IR injury by anoxia-reoxygenation (AR) *in vitro*

We next established a model of IR injury by mimicking reperfusion of anoxic cardiomyocytes. To do this, within the anoxic incubator the cells were first washed with anoxic buffer without the MCT1 inhibitor, then the cells were immediately removed from the anoxic incubator to room air while at the same time replacing the anoxic medium with fresh, oxygenated cell medium at 37 °C (Fig. 1a). This took ∼30 s, then the cells were removed to an aerobic 37 °C incubator. Replacing the anoxic medium with oxygenated medium lacking the MCT1 inhibitor replicates the rapid reperfusion with oxygenated blood that occurs during *in vivo* IR injury.

**Fig. 6:**
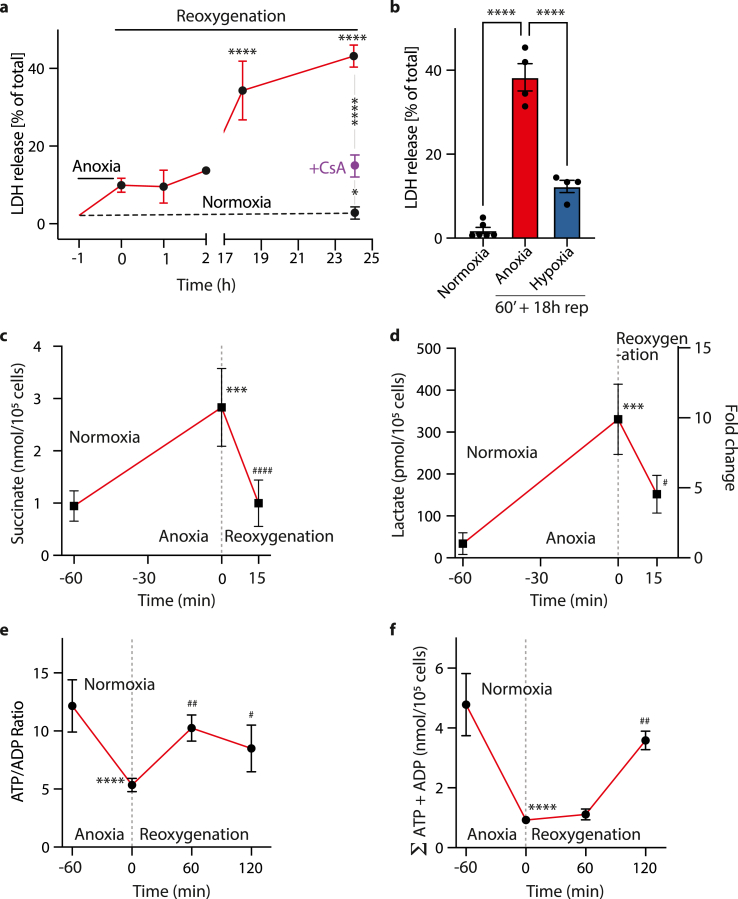
Cell metabolism and death after anoxia and reoxygenation. (a) Cell death was quantified after anoxia and reoxygenation at various stages. Primary adult mouse cardiomyocytes were maintained in a cell incubator in Tyrode's + 10 μM AR-C141990 either at 37 °C, 5% CO_2_ under normoxia for 25 h or exposed to anoxia for 1 h in an anoxic chamber at 37 °C, washed under anoxic conditions before oxygenated buffer without AR-C141990 was added and the cells were then incubated at 37 °C for up to 24 h. For some experiments 200 nM CyclosporinA was present. Cell death was determined by LDH activity measured in the cell supernatant. Results are presented relative to a positive control of cardiomyocytes lysed in 1% Triton-x-100. N = 3–5 biological replicates. Statistical analysis was performed via two-way ANOVA with Tukey's *post hoc* test, *P < 0.02, ****P < 0.0001. (b) Comparison of cell death after anoxia and reoxygenation, or hypoxia and reoxygenation. Primary adult mouse cardiomyocytes were maintained in a cell incubator in Tyrode's + 10 μM AR-C141990 either: at 37 °C, 5% CO_2_ under normoxia for 19 h; or exposed to anoxia for 1 h in an anoxic chamber at 37 °C, washed under anoxic conditions before oxygenated buffer without AR-C141990 was added and the cells were then incubated at 37 °C 5% CO_2_ under normoxia for 18 h; or exposed to hypoxia (0.1% O_2_) for 1 h in a hypoxic chamber at 37 °C, washed before oxygenated buffer without AR-C141990 was added and the cells were then incubated at 37 °C, 5% CO_2_ under normoxia for 18 h; Cell death was determined by LDH activity measured in the supernatant. Results are presented relative to a positive control of cardiomyocytes lysed in 1% Triton. N = 3–5 biological replicates. Statistical analysis was performed via one-way ANOVA with Tukey's *post hoc* test. ****P < 0.0001. (c–f) Primary adult mouse cardiomyocytes in Tyrode's + 10 μM AR-C141990 were exposed to anoxia for 1 h in an anoxic chamber at 37 °C, washed under anoxic conditions before oxygenated buffer without AR-C141990 was added and the cells were then incubated at 37 °C, 5% CO_2_ under normoxia for up to 2 h, as indicated. Control incubations under normoxia alone were for 75 min (c, d), or 3 h (e, f). Then cell pellets were analyzed at different time points and metabolite pools evaluated. (c) Succinate (nmol/10^5^ cells), n = 4–5 biological replicates, (d) l-Lactate in pmol/10^5^ cells on the left and as fold change to normoxia on the right y-axis, n = 3–4, (e) ATP/ADP ratio and (f) the sum of ATP and ADP nucleotide concentrations (nmol/10^5^ cells). n = 4–5 biological replicates. Statistical analysis was performed via one-way ANOVA with multiple comparisons and Tukey's *post hoc* test, Significance compared to the normoxic value ***P < 0.0005, ****P < 0.0001. Significance compared to the final anoxic timepoint (t = 0), ^#^P < 0.05, ^##^P < 0.01, ^####^P < 0.001.

To assess if rapid reoxygenation after anoxia caused cell damage akin to that of IR injury *in vivo*, we assessed cell death after AR by lactate dehydrogenase (LDH) release (Fig. 6a). This showed extensive cell death which was evident 18 h after reoxygenation, presumably due to the time taken for cells to rupture and release LDH. The addition of the inhibitor of the mitochondrial permeability transition pore Cyclosporin A (CsA) prevented cell death, measured as LDH release at 24 h after reoxygenation (Fig. 6a), consistent with induction of the mitochondrial permeability transition pore (MPTP) contributing to cell death. The cell death caused by reoxygenation after hypoxia (0.1% O_2_) was far less damaging than AR (Fig. 6b), further indicating that AR is a better model of IR injury than hypoxia/reoxygenation.

During IR injury *in vivo* the succinate and lactate levels rapidly return to normoxic values within minutes of reperfusion while in contrast, the ATP/ADP ratio and the adenosine nucleotide content take far longer to recover [[1], [2], [3], [4], [5]]. Upon reoxygenation of anoxic cardiomyocytes, succinate (Fig. 6c) and lactate (Fig. 6d) returned close to normoxic levels within 15 min of reoxygenation while the ATP/ADP ratio and the adenosine nucleotide content took far longer to recover (Fig. 6e and f). Thus, the AR cardiomyocyte model replicates many of the metabolic changes that occur during the development of cardiac IR injury *in vivo*.

### 2.3 Mitochondrial membrane potential during anoxia

**Fig. 7:**
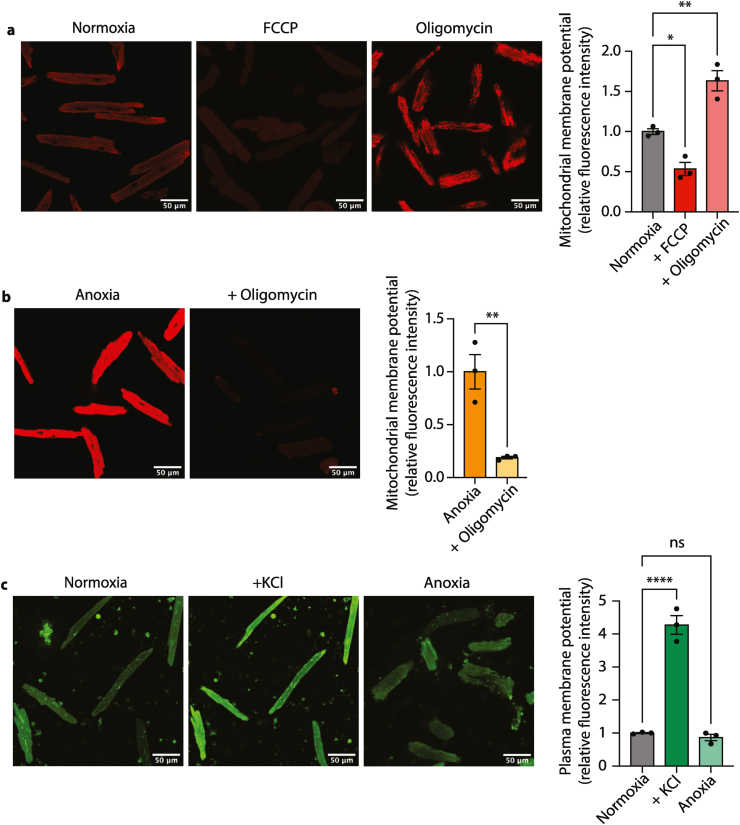
Mitochondrial and plasma membrane potentials during anoxia. (a) Mitochondrial membrane potential measurements during normoxia and 2 μM FCCP and 5 μM oligomycin. Illustrative confocal images and the bar chart show quantification of mitochondrial membrane potential. n = 3 biological replicates, statistical analysis was performed via one-way ANOVA with Tukey's post hoc test, *P < 0.04, **P < 0.003. (b) Mitochondrial membrane potential during anoxia (30 min) and anoxia + oligomycin after 10 min n = 3 biological replicates, statistical analysis was performed via one-way ANOVA with Tukey's *post hoc* test, **P < 0.003. (c) Plasma membrane potential measurements in normoxia, following the addition of 150 mM KCl and at 30 min anoxia. n = 3 biological replicates, statistical analysis was performed via one-way ANOVA with Tukey's *post hoc* test, ****P < 0.0001.

Despite the lack of oxygen for mitochondrial respiration, it is possible that the mitochondrial membrane potential (Δψ_m_) is maintained during ischemia through ATP hydrolysis and proton pumping by the F_o_F_1_-ATP synthase [18]. Therefore, we set out to assess Δψ_m_ during anoxia. To do this we monitored the accumulation of the Δψ_m_-dependent fluorescent probe tetramethylrhodamine (TMRM) under non-quenching mode, assessed by live-cell confocal microscopy [18] (Fig. 7). Control experiments with normoxic cardiomyocytes showed that TMRM fluorescence decreased when Δψ_m_ was dissipated using the uncoupler FCCP and increased when utilization of the Δψ_m_ for ATP synthesis was blocked with oligomycin (Fig. 7a). To monitor Δψ_m_ during anoxia we incubated cardiomyocytes under anoxic conditions, then sealed the culture flasks within the anoxic chamber in the presence or absence of oligomycin, and then transferred the flasks to the confocal microscope (Fig. 7b). Control experiments showed that this sealing procedure maintained anoxia for at least 10 min (Fig. S1). We observed anoxic accumulation of TMRM which was sensitive to oligomycin (Fig. 7b), consistent with generation of the Δψ_m_ through ATP hydrolysis by the F_o_F_1_-ATP synthase during anoxia [18]. The uptake of TMRM into mitochondria will also respond to changes in the plasma membrane potential (Δψ_p_) [[19], [20], [21]]. Therefore, we assessed if there were changes in Δψ_p_ during anoxia using the anionic anoxol dye ‘plasma membrane potential indicator’ (PMPI) obtained from the FLIPR Membrane Potential Assay kit (Molecular Devices) [[19], [20]]. Control experiments showed that in the presence of excess potassium the Δψ_p_ was abolished, hence changes in Δψ_p_ could be assessed in cardiomyocytes (Fig. 7c). The Δψ_p_ did not change in anoxia (Fig. 7c) indicating that our measurements of Δψ_m_ by TMRM fluorescence is not impacted by changes in Δψ_p_.

### 2.4 Changes in the redox state of NADH and CoQ during anoxia and upon reoxygenation

**Fig. 8:**
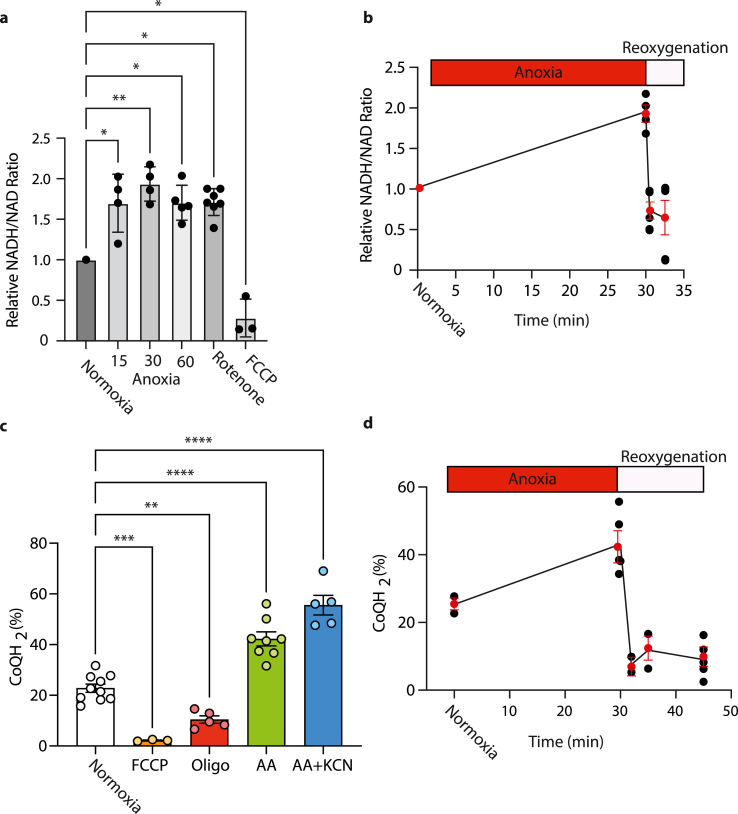
Redox states of the NADH/NAD^+^ and CoQH_2_/CoQ pools during anoxia and reoxygenation. In order to determine NADH pools and CoQ reduction state during anoxia, primary adult mouse cardiomyocytes were maintained either: in a cell incubator in Tyrode's + 10 μM AR-C141990 at 37 °C, 5% CO_2_ under normoxia for 2 h; or exposed to anoxia for 15, 30 or 60 min in an anoxic chamber at 37 °C and lysed under anoxia; or washed under anoxic conditions before oxygenated buffer without AR-C141990 was added and the cells were then incubated at 37 °C for up to 15 min to induce reoxygenation. For cell lysis, buffer was rapidly removed and the cells scraped down in lysis buffer at 4 °C. n = 3–7 biological replicates, statistical analysis was performed via one-way ANOVA with multiple comparisons and Tukey's *post hoc* test, *P < 0.05, **P < 0.007, ***P = 0.0001, ****P < 0.0001. (a) Cardiomyocytes were incubated under normoxic conditions with no additions, or with rotenone 1 μM or FCCP 2 μM, or under the indicated times of anoxia. Then the whole cell NADH/NAD ratio was determined and is expressed relative to that of anoxic cells. (b) Cardiomyocytes were incubated under normoxic conditions or for 30 min of anoxia alone or with the indicated times of reoxygenation. Then the whole cell NADH/NAD ratio was determined and is expressed relative to that of normoxic cells. (c) Cardiomyocytes were incubated under normoxic conditions with no additions, or with FCCP (2 μM), or oligomycin (2 μM), or antimycin (2 μM) or antimycin (2 μM) and KCN (2 mM). Then the reduction state of the CoQ pool was determined. (d) Cardiomyocytes were incubated under normoxic conditions, or under anoxia alone, or under anoxic conditions followed by various times of reoxygenation. Then the reduction state of the CoQ pool was determined.

The flux of electrons within the mitochondrial inner membrane during ischemia and reperfusion is central to the generation of mitochondrial superoxide and hydrogen peroxide during IR injury. Hence, we measured how the two key mitochondrial electron carriers, the NADH/NAD^+^ and the CoQH_2_/CoQ pools, are altered during anoxia and reoxygenation. We measured the NADH/NAD^+^ ratio using an end point enzymatic assay which enabled us to measure the changes in the whole cell ratio following rapid (<10 s) quenching of the cells after incubation. In control experiments, the whole cell NADH/NAD^+^ ratio increased upon inhibition of complex I with rotenone and decreased upon uncoupling of mitochondria with FCCP (Fig. 8a), consistent with this assay accurately reporting relative changes in the combination of the mitochondrial and cytosolic NADH/NAD^+^ ratios within cells. During anoxia for 15–60 min the ratio increased compared to normoxia (Fig. 8a). Upon reoxygenation after anoxia the ratio very rapidly decreased to a level below that of normoxia (Fig. 8b).

Next, we assessed the CoQH_2_/CoQ ratio using an LC-MS/MS method we recently developed [22], which enables the ratio to be determined by rapidly (<10 s) quenching the cell incubation. In normoxic cardiomyocytes this ratio increased upon inhibition of respiration with antimycin and decreased upon uncoupling (Fig. 8c). Next, we assessed how the modifiable CoQH_2_/CoQ ratio altered in response to anoxia and found that it increased after 30 min anoxia (Fig. 8d). Subsequent reoxygenation rapidly oxidized the CoQ pool which then stayed relatively oxidized after reoxygenation for up to 15 min (Fig. 8d).

### 2.5 Superoxide generation, hydrogen peroxide production and oxidative damage upon reoxygenation

**Fig. 9:**
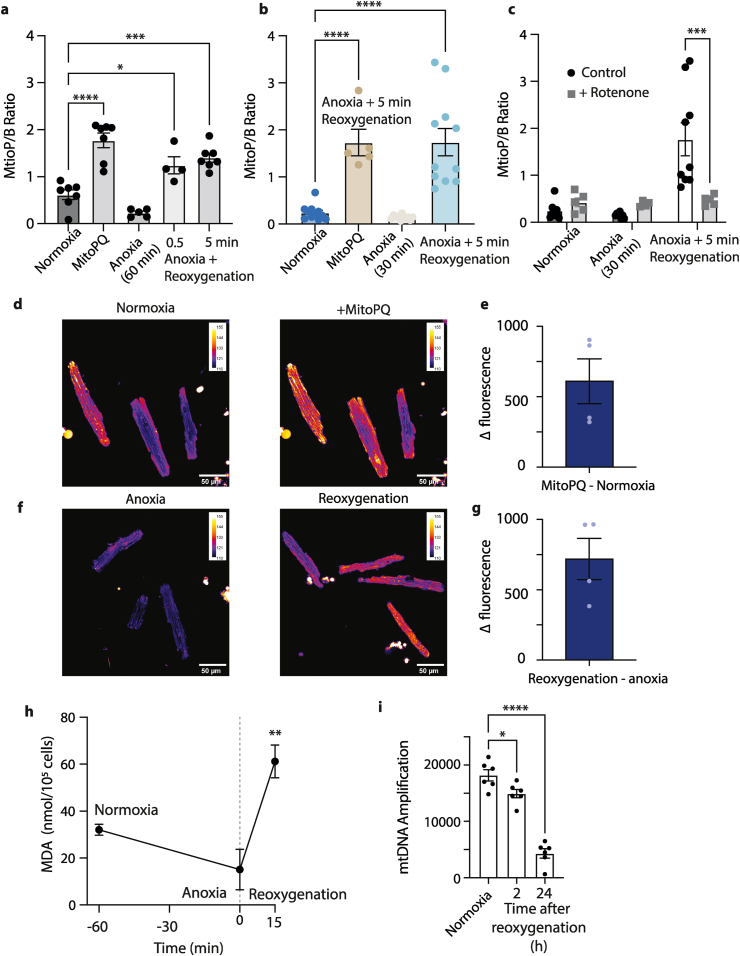
Mitochondrial superoxide and hydrogen peroxide production after anoxia and reoxygenation. Primary adult mouse cardiomyocytes were maintained as controls in a cell incubator, or subjected to 10 min 2 μM MitoPQ treatment at 37 °C, or to 1 h of anoxia at 37 °C in Tyrode's + 10 μM AR-C141990. Cells were reoxygenated in Tyrode's buffer. (a–c) the MitoP/MitoB ratio was determined under the following conditions. (a) normoxia for 65 min, anoxia for 60 min or anoxia for 60 min followed by 0.5 or 5 min reoxygenation. (b) normoxia for 30 min, anoxia for 30 min or anoxia for 30 min followed by 5 min reoxygenation. (c) normoxia for 35 min, anoxia for 30 min or anoxia for 30 min followed by 5 min reoxygenation ± rotenone. Rotenone was added 10 min prior to the end of anoxia. (d–g) Assessment of MitoSOX fluorescence showing typical confocal images with a scale indicating grey value along with quantification of changes in fluorescence. (d) shows normoxic cells incubated with MitoSOX for 15 min and then measured again 5 min after the addition of 5 μM MitoPQ, with the quantification of the fluorescence change upon addition of MitoPQ shown in (e). (f) shows the MitoSOX fluorescence in a sealed anoxic chamber following 30 min anoxia. In (f) is also shown the anoxic sample after it is opened to the air by removing the sealed lid and measuring MitoSOX fluorescence 2 min later, with quantification being shown in (g). (h) Measurement of MDA in cells after exposure to reoxygenation. (i) mtDNA damage was investigated using qPCR in DNA extracted from whole cell lysates. Decreasing amounts of DNA amplification indicate increasing damage on mtDNA. n = 3–11 biological replicates, statistical analysis was performed via one-way ANOVA or two-way ANOVA for rotenone experiments (c) with multiple comparisons and Tukey's *post hoc* test, *P < 0.02, **P < 0.002, ***P = 0.0001, ****P < 0.0001.

To determine whether reoxygenation of anoxic cardiomyocytes led to mitochondrial superoxide production, which would be rapidly dismutated to hydrogen peroxide, we next measured the production of these ROS and oxidative damage upon reoxygenation (Fig. 9). To assess mitochondrial hydrogen peroxide production, we measured the MitoP/MitoB ratio by LC-MS/MS, which should increase in response to elevated hydrogen peroxide [23]. As a positive control we used the mitochondria-targeted redox cycler MitoPQ [24], which increased the MitoP/MitoB ratio (Fig. 9a and b). This ratio was low after 30 or 60 min anoxia (Fig. 9a and b). Reoxygenation led to an increase of the MitoP/MitoB ratio upon 30 s of reoxygenation after 60 min anoxia and this level did not increase further when measured 5 min after reoxygenation, following 30 or 60 min anoxia (Fig. 9a and b). Treatment of cardiomyocytes with the complex I inhibitor rotenone did not markedly increase the MitoP/MitoB ratio during normoxia or anoxia (Fig. 9c). However, rotenone blocked the increase in the MitoP/MitoB ratio after reoxygenation (Fig. 9c).

To corroborate the mass spectrometry analysis of mitochondrial superoxide/hydrogen peroxide generation, we measured the oxidation of MitoSOX by confocal microscopy. Control experiments showed that MitoPQ oxidized MitoSOX leading to increased fluorescence intensity (Fig. 9d). The quantification of this increase is shown in Fig. 9e. Cardiomyocytes that were exposed to anoxia and incubated with MitoSOX under these conditions did not show any significant increase in fluorescence over 10 min (Fig. S1). When we opened the flask to room air there was a dramatic increase in MitoSOX fluorescence when measured ∼2 min after reoxygenation (Fig. 9f and g).

To see if the elevated superoxide/hydrogen peroxide production led to oxidative damage, we measured lipid peroxidation via the formation of thiobarbituric acid reactive species (TBARS) [25] which increased rapidly upon reoxygenation (Fig. 9h). Next, we assessed mitochondrial oxidative damage by measuring disruption to mtDNA caused by adducts or strand breaks by a quantitative PCR assay (Fig. 9i) [26]. This showed that mtDNA was damaged 2 h after reperfusion consistent with elevated mitochondrial oxidative damage upon reoxygenation. The mtDNA damage increased further 24 h after reoxygenation in association with cell death. Together these findings are consistent with an increase in mitochondrial superoxide and hydrogen peroxide production upon reoxygenation.

## 3 Conclusions

We have established a cell model of cardiac IR injury using adult murine cardiomyocytes that replicates many key aspects of *in vivo* IR injury. In particular, our model uses anoxia, as opposed to hypoxia, and inhibition of lactate efflux to replicate ischemia *in vivo*. Anoxia is a critical, and often overlooked, experimental consideration because oxygen levels of ∼0.1% used during hypoxia readily sustain mitochondrial oxidative phosphorylation due to the low K_m_ for O_2_ of cytochrome *c* oxidase. The use of pharmacological MCT1 inhibition is also important as *in vitro* the efflux of lactate into the cell medium will enable cells to maintain a lower NADH/NAD ^+^ ratio and thereby sustain glycolytic flux to a far greater extent than possible during ischemia *in vivo*. Importantly, we demonstrated our anoxic *in vitro* model reproduces the metabolic hallmarks of *in vivo* ischemia: succinate and lactate accumulation as well as a decrease in the ATP/ADP ratio that was associated with the rapid loss of adenine nucleotides. In contrast, incubation of cardiomyocytes under hypoxic conditions (0.1% O_2_) with MCT1 inhibition did not replicate *in vivo* cardiac ischemia. Anoxic incubation with MCT1 inhibition also led to the expected increase of the cellular NADH/NAD^+^ and CoQH_2_/CoQ ratios. It was also possible to transfer the cells to a confocal microscope under anoxic conditions to assess the live-cell Δψ_p_ and Δψ_m_. This enabled us to show that the Δψ_m_ is maintained during ischemia by ATP hydrolysis and proton pumping by the F_o_F_1_-ATP synthase, consistent with the *in vivo* situation [18]. The Δψ_p_ was unaffected by short periods of anoxia, a key and often omitted control that is required to interpret measurements of Δψ_m_ by the accumulation of TMRM [[19], [20], [21]]. In summary, this anoxic cardiomyocyte model will be of great utility in modeling and investigating the metabolic and mitochondrial changes that occur during ischemia and are difficult to assess *in vivo*.

We also explored whether this cell model could replicate key markers of cardiac IR injury. We found we could mimic many facets of IR injury by rapidly reoxygenating the cells following a period of anoxia. We could use this model to confirm rapid oxidation of NADH, CoQ and succinate upon reoxygenation. This led to an increase in the production of hydrogen peroxide, oxidative damage and cell death upon reoxygenation, thereby modeling the pathological changes that occur during IR injury *in vivo*. In summary, we have developed an anoxic cell model of cardiac ischemia that can be used to model mitochondria changes during ischemia and reperfusion.

## 4 Materials and methods

### 4.1 Animals

All experiments were performed under UK Home Office Licences and conducted according to the Animals Scientific Procedures Act 1986 (UK) and directive 2010/63/EU of the European Parliament guidelines on the protection of animals used for scientific purposes. All experiments were approved by the Institutional Animal Welfare and Ethical Review Body. C57BL/6J male mice (∼25 g, 8–12 weeks old) were from Charles River, UK.

### 4.2 Isolation of adult primary mouse cardiomyocytes

Adult primary mouse cardiomyocytes were isolated as described previously [27,28] C57/BL6J mice were culled by cervical dislocation before rapidly excising the heart and cannulating via the aorta in ice-cold, sterile perfusion buffer (in mM: NaCl (113), KCl (4.7), KH_2_PO_4_ (0.6), Na_2_HPO_4_ (0.6), MgSO_4_·7H_2_O(1.2), NaHCO_3_ (12), KHCO_3_ (10), HEPES sodium salt (0.922), taurine (30), 2,3-butanedione monoxime (10) and glucose (5.5)). Hearts were retrogradely perfused for 5 min with perfusion buffer (37 °C) to clear residual blood, then the hearts were digested by perfusing digestion buffer (30 mL perfusion buffer supplemented with 5 mg Liberase (Roche, UK) and 12.5 μM CaCl_2_) for 20 min. After digestion, the heart was removed from the cannula and carefully broken apart with tweezers and gentle pipetting in 4 mL digestion buffer. The cell suspension was transferred to a 15 mL centrifuge tube before making up to 10 mL with stop buffer (37 °C) (10% [v/v] fetal bovine serum [FBS] (in perfusion buffer) and allowed to pellet by gravity for 10 min at RT. After pelleting, the supernatant was removed and the cells were resuspended in sequentially increasing Ca^2+^ concentrations (62 μM, then 212 μM, then 1 mM) in 5 mL stop buffer. Cells were subsequently resuspended in M199 media (Gibco, Thermo Fisher Scientific) supplemented with 2 mM l-carnitine, 5 mM creatine, 5 mM taurine, 25 μM blebbistatin, 100 IU/mL penicillin and 100 IU/mL streptomycin and plated either at (1 × 10^5^ cells/dish) on 52 mm glass dishes or at 3 × 10^4^ on 31 mm glass dishes, which all had been laminin-coated for 4 h (0.1 mg/mL from Engelbreth-Holm-Swarm murine sarcoma basement membrane; Sigma Aldrich, UK) before plating. Cell preparations were assessed for morphology and viability by Trypan Blue staining or propidium iodide exclusion as well as via LDH release assays. This showed a combination of rod-shaped and round cells visible on a 4x brightfield microscope immediately after plating. On average, cardiomyocyte isolation led to ∼60–65% rod shaped cells and 35–40% round cells initially, with 97% of the round cells being washed off after changing the medium 4 h after plating, leaving an essentially pure population of rod-shaped cardiomyocytes. No increased LDH release over 24 h of cell culture indicate no excessive cell death.

### 4.3 Anoxic cardiomyocyte incubations

Anoxic incubations were carried out using an anaerobic chamber (∼0.1–10 ppm O_2_; Belle Technologies, UK). Equipment and solutions were degassed for between 30 min and up to overnight in the transfer compartment of the anoxic chamber before the experiment was performed. Experiments were performed in Tyrode's buffer (in mM: NaCl (137), KCl (5.4), MgCl_2_ (0.4), HEPES (10), Glucose (10), CaCl_2_ (1), pH 7.4). The MCT1 inhibitor AR-C141990 (Tocris, Bio-Techne) was used at a concentration of 10 μM in Tyrode's buffer.

Cardiomyocytes were washed once with either 2 mL Tyrode's buffer (52 mm dishes) or 1 mL Tyrode's buffer (31 mm dishes) (37 °C) before the same volume of fresh Tyrode's buffer was added to each dish. Anoxia was induced for different time points by placing dishes in the anaerobic chamber on a 37 °C heat block before the cells were lysed under anoxia, transferred to Eppendorf tubes and snap frozen on dry ice. Cells were reoxygenated by removing them from the anaerobic chamber, replacing buffer with fresh, oxygenated Tyrode's buffer and incubating (37 °C) for various times before lysing cells for subsequent analysis. Metabolite levels in cells were normalized to the number of cells initially plated in the culture dish.

For live cell imaging, cells were plated at 3 × 10^4^ cardiomyocytes per 27 mm on Nunc glass bottom dishes (Thermo scientific, Cat no 150680) and stained either under normoxic conditions or in the anoxic glove box in Tyrode's buffer supplemented with MCT1 inhibitor AR-C141990 at 10 μM as well as various dyes as described below. In order to maintain an anoxic environment, the lid of the dish was layered with Blu Tack™ around the circumference while the edge of the bottom dish was also layered in this way. This enabled the lid to be tightly sealed while the cells were in the anoxic chamber. This procedure enabled the transport of the cell incubation from the anoxic incubator to the confocal microscope while maintaining an anoxic environment and performing live cell imaging.

### 4.4 Hypoxic cell incubations at 0.1% O_2_

In order to incubate cells hypoxically at oxygen concentrations accurately monitored at levels as low as 0.1% O_2_, the BMG Labtech CLARIOstar *Plus* plate reader with gas control function was utilised. The gas control module enables the generation of an 96.9% N_2_, 3% CO_2_ and 0.1% O_2_ at 37 °C in a small sealed environment in the machine's plate chamber. The gas exchange was controlled via ventils at the incubator and monitored via the in-built gas sensors. Cardiomyocytes were plated on laminated 6-well plates (Costar) at 10^5^ cells/well for hypoxia experiments. Sample retrieval and cell lysis was performed by removing the plate from the sealed hypoxic environment, rapidly removing the cell medium and immediately lysing the cells in appropriate lysis buffer. This was performed on ice within 20 s after removal of the plate from the hypoxic environment.

### 4.5 Succinate extraction and analysis

Succinate from adult primary cardiomyocytes (10^5^ cells) was extracted in 500 μL MS extraction buffer (50% [v/v] methanol, 30% [v/v] acetonitrile and 20% [v/v] H_2_O), supplemented with 1 nmol of [^13^C_4_]-succinate (Sigma Aldrich, UK) in a pre-chilled Eppendorff tube and agitated in the cold (1400 rpm, 15 min, 4 °C; Thermo Fisher Scientific, UK) before incubating (−20 °C, 1 h). The samples were centrifuged (17,000×*g*, 10 min, 4 °C) the supernatant transferred to a new tube and recentrifuged. The clear supernatant was transferred to MS vials and stored at −80 °C until analysis.

LC-MS/MS analysis of succinate was performed as described [29] using an LCMS-8060 mass spectrometer (Shimadzu, UK) with a Nexera X2 UHPLC system (Shimadzu, UK). Samples were stored in a refrigerated autosampler (4 °C) upon injection of 5 μl into a 15 μl flowthrough needle. Separation was achieved using a SeQuant® ZIC®-HILIC column (3.5 μm, 100 Å, 150 × 2.1 mm, 30 °C column temperature; MerckMillipore, UK) with a ZIC®-HILIC guard column (200 Å, 1 × 5 mm). A flow rate of 200 μl/min was used with mobile phases of Buffer A: 10 mM ammonium bicarbonate and Buffer B: 100% acetonitrile. A gradient of 0–0.1 min, 80% MS buffer B; 0.1–4 min, 80%–20% B; 4–10 min, 20% B, 10–11 min, 20%–80% B; 11–15 min, 80% B was used. The mass spectrometer was operated in negative ion mode with multiple reaction monitoring (MRM) and spectra were acquired using Labsolutions software (Shimadzu, UK), with compound quantities calculated from relevant standard curves in MS extraction buffer and comparing against [^13^C_4_]-succinate internal standard.

### 4.6 Lactate assay

Lactate was measured in cell supernatants or in cell pellets. For analysis of the intracellular lactate content, the cardiomyocytes were washed and lysed in 200 μL assay buffer/10^5^ cells by scraping them down and repeating three freeze/thaw cycles and vigorous vortexing in between these cycles. The samples were centrifuged for 2 min at 14,000 g at 4 °C and the supernatant kept for deproteinization. Therefore, 25 μL 5 M perchloric acid were added to each tube, followed by 47 μL of 3 M potassium hydroxide to neutralize the samples. After centrifugation for 5 min at 14,000 g at 4 °C, the supernatant was used to determine lactate concentrations using the colorimetric Abcam Lactate Assay Kit (ab65331). According to the manual instructions, a standard curve was prepared via serial dilution of the kit's standard concentrate in assay buffer. 50 μL of each standard, blank or sample were pipetted into a CoStar 96- well plate. Then, a reaction master mix was prepared containing 46 μL assay buffer, 2 μL substrate mix and 2 μL enzyme mix per reaction. 50 μL of this master mix were added to each well and the reaction was incubated for 30 min at 400 rpm at room temperature. The output was measured on a microplate reader (CLARIOstar *PLUS* from BMG Labtech) at OD 450 nm.

### 4.7 ATP/ADP assay

ATP and ADP concentrations were measured using a Luciferase based assay [30]. 10^5^ cells were scraped down in 1 mL ice-cold perchloric acid extractant (3% v/v HClO_4_, 2 mM Na_2_EDTA, 0.5% Triton X-100). Samples, ATP and ADP standards (400 μL) were pH neutralized using a potassium hydroxide solution (2 M KOH, 2 mM Na_2_EDTA, 50 mM MOPS), vortexed until formation of a white precipitate (KClO_4_), then centrifuged (17, 000×*g* for 1 min at 4 °C). For ADP measurements, 250 μL neutralized sample supernatant was mixed with 250 μL ATP sulfurylase assay buffer (20 mM Na_2_MoO_4,_ 5 mM GMP, 0.2 U ATP sulfurylase (New England Biolabs), in Tris-HCl buffer (100 mM Tris-HCl, 10 mM MgCl_2_ (pH 8.0)) to 250 μL), incubated for 30 min at 30 °C with shaking (500 rpm), heated at 100 °C for 5 min and then cooled on ice. Standards (100 μL), samples for ATP measurement (100 μL) or samples for ADP measurement (200 μL) (in duplicate) were added to 400 μL Tris-acetate (TA) buffer (100 mM Tris, 2 mM Na_2_EDTA, 50 mM MgCl_2,_ pH 7.75 with glacial acetic acid) in luminometer tubes. 10 μL pyruvate kinase solution (100 mM PEP, 6 U pyruvate kinase suspension (Sigma #P1506)) were added to one set of samples for ADP measurement and incubated for 30 min at 25 °C in the dark to convert ADP to ATP. The other duplicate tube (without addition of pyruvate kinase solution) served as an ATP ‘blank’ value. The samples were then all assayed for ATP content in a Berthold AutoLumat Plus luminometer by addition of 100 μL Luciferase/Luciferin Solution (7.5 mM DTT, 0.4 mg/mL BSA, 1.92 μg luciferase/ml (SIGMA #L9506), 120 μM D-luciferin (SIGMA #L9504), made in TA buffer (25% v/v glycerol)), delivered via auto injection, protected from light. Bioluminescence of the ATP- dependent luciferase activity was measured 3X for 1 min post injection and the data quantified against standard curves.

### 4.8 Cell death assay

1000 μL supernatant were taken off from control cells, ischemic cells or ischemic-reperfused cells at 10^5^ cells/plate. They were either not treated, treated or treated with a vehicle (DMSO). The supernatant was spun for 10 min at 1000×*g* at 4 °C and stored in a fresh Eppendorff tube at −80 °C. For analysis of cell death, lactate dehydrogenase was measured using the Roche (Sigma-Aldrich) cytotoxicity kit (Cat. No. 11,644,793 001) utilizing the reaction of LDH with its substrate lactate via reduction of NAD^+^ visible in form of a red color which can be measured on a microplate reader. 100 μL supernatant were transferred to a CoStar 96- well plate. Each plate also contained a positive control of cell lysate of 100,000 cells in 1% Triton-X100 and appropriate blanks. The substrate solution was freshly prepared with 11.25 mL of dye solution A, which was mixed with 250 μL of catalyst solution B. 100 μL of this substrate solution were added to each sample. The plate was incubated at room temperature for 15 or 30 min before absorbance at 450 and 620 nm was measured on a microplate reader (CLARIOstar *PLUS* from BMG Labtech).

### 4.9 Oxidative damage assays

Oxidation of mtDNA was assayed by quantitative polymerase chain reaction (PCR) using two targets: a short target (≥200 bp), to control for the mtDNA copy number, and a long target (∼10 kbp), for which reduced amplification corresponds to oxidative DNA damage^29,30^. DNA was isolated from 50 fresh or thawed pancreatic islets using GenElute™ Mammalian Genomic DNA Miniprep Kit (Sigma-Aldrich) according to manufacturer's instructions and DNA stored at −20 °C until the assay. Prior to the assay, DNA concentration in the thawed samples was measured using NanoDropTM 8000 Spectrophotometer (ThermoFisher Scientific) and samples were diluted to 3 ng/mL. The PCR mastermix contained 200 pM dNTPs (TaKaTa), 200 pM forward primer (Sigma, 5′-GCC AGC CTG ACC CAT AGC CAT AAT-3′), 200 pM reverse primer (Sigma, 5′- GCC GGC TGC GTA TTC TAC GTT A -3′ for short target, 5′-GAG AGA TTT TAT GGG TGT AAT GCG G-3′ for long target), 100 ng/mL BSA (New England Biolabs) and 1 mM Mg(OAc)_2_ (TaKaRa) in buffer (TaKaRa). Reaction was performed in PCR tubes and each reaction consisted of 5 μL (15 ng) of DNA template from samples, 35 μL of PCR mastermix and 1 U of LA Taq polymerase (TaKaRa). Reactions were performed in duplicates, including non-template control and linear amplification control. Reactions were then performed using 22 cycles for short target and 25 cycles for long target. PCR products were quantified using the Quant-iT™ PicoGreen™ dsDNA Assay Kit (ThermoFisher Scientific) according to manufacturer's instructions and the size of the PCR product was checked on a 0.8% agarose gel. For the analysis in Excel, all samples were corrected to the non-template control and normalized to amplification of the short target.

### 4.10 Thiobarbituric acid reactive substances (TBARS) assay

Malondialdehyde (MDA) standard was prepared by hydrolysing 1,1,3,3- tetraethoxypropane (TEPP). For standard curves, 10 mM TEPP was prepared in 1% v/v sulfuric acid and stored at 4 °C overnight. 10 mM TEPP was further diluted with deionized water to create standards ranged from 2000 nmol TEPP to 31.25 nmol TEPP. Standards were made up fresh for each assay. 30 μl of 2% butylated hydroxytoluene and 100 μL of 35% perchloric acid and thiobarbituric acid (mixed at equal volume) were added to each cell plate. A cell scraper was used to collect cells from a glass plate and the lysate was transferred to a fresh tube. After brief spinning, lysates were incubated for 15 min at 100 °C. Once cell lysates were cooled to room temperature, the entire lysate was transferred to a tube containing 1.5 mL of butan-1-ol and 1.5 mL of deionized water. The tube was vortexed vigorously and allowed 2 layers to form. 250 μL of butan-1-ol was collected from the top layer and transferred in triplicate to a microplate before fluorescence intensity was measured (excitation at 515 nm and emission at 553 nm). For positive controls, extraction was performed in the same way as samples by adding 35% perchloric acid and thiobarbituric acid, except 2% butylated hydroxytoluene was not added. 50 μl of *tert*-butyl hydroperoxide and the final concentration of 50 μmol iron (II) chloride were added subsequently. The infusion of *tert*-butyl hydroperoxide induces a rapid, sustained release of TBARS. Once samples were briefly spun down, positive controls were incubated at 37 °C for 1 h followed by 15 min incubation at 100 °C. The same procedures as with samples were performed with standards afterward. This method was adapted from Ref. [25].

### 4.11 CoQ extraction and evaluation

Cells (3 × 10^4^) were plated on glass dishes and subjected to normoxia, anoxia or treated with inhibitors. The inhibitors were added at the following concentrations: 2 μM FCCP, 2 μM antimycin A, 2 mM KCN. Cells were incubated with inhibitors for 10 min at 37 °C, washed with PBS and scraped in 200 μL fresh PBS on ice prior to CoQ extraction. To extract CoQ from cells, the cell suspension in 200 μL PBS was rapidly transferred to ice cold extraction solution (200 μL acidified methanol and 300 μL of hexane) followed by immediate vortexing. The upper, CoQ-containing hexane layer was separated by centrifugation (5 min, 17,000×*g*, 4 °C) and then dried down in 1 mL glass mass spectrometry vials (186005663CV, Waters) under a stream of N_2_. Dried samples were then resuspended in 300 μL methanol containing 2 mM ammonium formate, overlaid with argon and stored at −20 °C until analysis.

LC-MS/MS analyses were carried out as described [22] using an I-Class Acquity LC system attached to a Xevo TQ-S triple quadrupole mass spectrometer (Waters) using published parameters [22]. Samples were kept at 8 °C prior to injection by the autosampler of 2–10 μL into a 15 μL flow-through needle and RP-HPLC at 45 °C using an Acquity C18 column (2.1 × 50 mm, 1.7 μM; Waters). Mobile phase was isocratic 2 mM ammonium formate in methanol run at 0.8 mL/min over 3 min. For MS analysis, electrospray ionization in positive ion mode was used as described above. Multiple reaction monitoring in positive ion mode was used for compound detection. Transitions used for quantification were: CoQ_9_, 812.9 > 197.2; CoQ_10_, 880.9 > 197.2; CoQ_9_H_2_, 814.9 > 197.2; CoQ_10_H_2_, 882.9 > 197.2. Samples were quantified using MassLynx 4.2 software to determine the peak area for CoQ_9_, CoQ_10_, CoQ_9_H_2_, CoQ_10_H_2_. As the predominant CoQ species in mice are CoQ_9_ and CoQ_9_H_2_ reported data are for these.

### 4.12 NAD^+^ and NADH quantification assay

In order to measure NAD^+^ and NADH concentrations in adult cardiomyocytes, the NAD^+^/NADH Quantification kit from Promega (G9072) was used linking NAD cycling to luciferase/luciferin dependent light production measured via luminescence. 10^4^ cells were subjected to various times of anoxia and reoxygenation before lysis in 50 μL PBS +50 μL 1% DTAP in 0.2 M NaOH.

To run the assay, standard curves of NAD^+^ and NADH (Sigma-Aldrich) between 0 and 2 mM were prepared freshly in lysis buffer and pipetted in duplicates in 25 μL total volumes onto a white 96 well plate (Costar). The samples were spun for 10 min at 17,000 g at 4 °C. 25 μL of the supernatant were pipetted onto the plate in duplicates for NADH and for NAD^+^ quantification. To determine NAD^+^ specifically, 12.5 μL 0.4 M HCl was added to these well and the plates incubated for 15 min at 60 °C. After equilibration to room temperature, 12.5 μL 0.4 M HCl was added to the NADH wells as well, followed by 12.5 μL 0.5 M Tris added to all wells leading to a final well volume of 50 μL.

A master mix containing 50 μL Reagent Buffer and 0.25 μL each of Reductase, Substrate, NAD Cycling Enzyme, as well as 1.25 μL NAD^+^ Cycling Substrate per well was prepared according to manufacturer's instructions. After addition of the 50 μL master mix to each well, Ultra-Glo luminescence was measured according to manufacturer's instructions using a BMG Labtech CLARIOstar *Plus* plate reader with luminometer function. NADH and NAD + concentrations were determined via the standard curve.

The residual sample supernatant was used in duplicates for protein quantification via bicinchoninic acid assay to normalize the NADH/NAD^+^ levels for sample protein content. Therefore, a working solution was made by mixing reagent A (EMD Millipore Corp., 3483636) and reagent B (Thermo scientific, UJ291929) at a 50:1 ratio. 10 μL of a bovine serum albumin standard (between 0 and 2 μg/μL) or sample were added per well in duplicates. 190 μL of the working solution was added to each well and the microplate was incubated for 30 min at 37 °C before absorbance was measured at 562 nm and the protein concentration in each sample was determined using the standard curve.

### 4.13 Laser scanning confocal microscopy measurements

All images were acquired using a Zyla 4.2 PLUS sCMOS camera attached to an Andor DragonFly 500 confocal spinning disk mounted on a Nikon Eclipse TiE microscope using a CFI Plan Apochormat lambda 40 × oil immersion objective. Images were acquired using the Fusion user interface (Andor) and analyzed using Fiji ImageJ. Ten regions of interest (ROI) of 11.76 μm × 11.76 μm were selected per biological replicate and the integrated density of the ROIs was used to quantify fluorescence intensity.

### 4.14 Mitochondrial membrane potential and MitoSOX measurements

Adult primary cardiomyocytes were incubated in 10 nM tetramethylrhodamine methyl ester perchlorate (TMRM) (Sigma) in M199 media for 20 min, 37 °C. Cells were washed with PBS once and the media was replaced with Tyrode's buffer containing 10 nM TMRM. To determine that TMRM was in non-quenching mode, cells were treated with 5 μ M oligomycin, 2 μ M Carbonyl cyanide-*p*-trifluoromethoxyphenylhydrazone (FCCP) or ethanol, as a vehicle control for 5 min and images were taken before and 5 min after treatment. Images were acquired during normoxia and after 30 min anoxia. Oligomycin sensitivity of the mitochondrial membrane potential during anoxia was tested by treating the cardiomyocytes with 5 μ M oligomycin for 5 min in the anoxic chamber before the dishes were sealed. All images using TMRM to measure mitochondrial membrane potential were acquired using 100 ms exposure, 10% laser intensity and excitation/emission 561/620 nm. Adult primary cardiomyocytes were incubated in 10 μ M MitoSOX for 15 min, 37 °C. Addition of 5 μM MitoPQ for 10 min was used as a positive control for mitochondrial superoxide/hydrogen peroxide production. MitoSOX was added to cells in the anoxic chamber 15 min before the dishes were sealed to monitor mitochondrial superoxide/hydrogen peroxide production during anoxia and then upon reperfusion. All images using MitoSOX to measure mitochondrial superoxide/hydrogen peroxide production were acquired using 200 ms exposure, 20% laser intensity and excitation/emission 561/620 nm.

### 4.15 Plasma membrane potential measurements

Component A explorer format from FLIPR® membrane potential assay kit (Molecular Devices, catalog no. R-8042), referred to as plasma membrane potential indicator (PMPI) was resuspended in 10 mL water and 10 μL of this stock was added to 1 mL Tyrode's buffer. The cardiomyocytes were imaged immediately. To assess changes in plasma membrane potential, images were taken immediately following the addition of 200 μL 150 mM KCl. PMPI was added to cells in the anoxic chamber before the dishes were sealed and just prior to imaging to monitor the plasma membrane potential during anoxia. Images were acquired using 300 ms exposure, 50% laser intensity and excitation/emission 514/530 nm.

### 4.16 Cell incubation of MitoB and LC-MS/MS determination of MitoB and MitoP

3 × 10^4^ adult cardiomyocytes were plated on laminated glass dishes as described before. Before starting treatment, the cells were incubated with 5 μM MitoB stock solution diluted in Tyrode's buffer for 30 min at 37 °C. Cells were washed and transferred into 1 mL of Tyrode's buffer supplemented with 10 μM MCT1 inhibitor AR-C141990 (Tocris) before being subjected to anoxia and/or reoxygenation for various timepoints, or to 5 μM MitoParquat for 10 min at 37 °C. After treatment, sample medium was rapidly removed and samples lysed in 200 μL MitoP/B extraction buffer A (60% (v/v) MS-grade acetonitrile (Romil, UK), 0.1% MS-grade formic acid (Merck, UK), 39.9% MS-grade water (Thermo Fisher Scientific, UK)) and 10 μL internal standard containing 100 pmol *d*_*15*_-MitoB and 50 pmol *d*_*15*_-MitoP. After incubation for 30 min at 4 °C, samples were then centrifuged (17,000 g, 10 min) and the supernatant transferred to a 96 well filter plate. The filtered supernatant was transferred to fresh 1.5 mL microcentrifuge tubes and dried in a speed vac overnight (40 °C). Dried samples were resuspended in 250 μL MitoP/B extraction buffer B 20% (v/v) MS-grade acetonitrile (Romil, UK), 0.1% MS-grade formic acid (Merck, UK), 79.9% MS-grade water (Thermo Fisher Scientific, UK), vortexed for 5 min and centrifuged (17,000 g, 10 min). The supernatant was then transferred to MS vials and stored at −20 °C until analysis.

A Waters Xevo TQ-S triple-quadrupole mass spectrometer was used for MS analysis as described previously [23]. Electrospray ionization was in positive ion mode; parameters were as follows: capillary voltage, 3.2 kV; cone voltage, 79 V; ion source temperature, 150 °C; collision energy, 45 V. Nitrogen and argon were used as the curtain and the collision gases, respectively. LC-MS/MS analyses were carried out using a front end I-class Aquity LC system (Waters). Samples were cooled and 2 mL injected using a 15 mL flow-through needle and RP-HPLC was carried out at 30 °C using an Acquity UPLC BEH C18 1 μm, 1 3 50 mm column (Waters). Buffers were 5% (v/v) ACN/0.1% (v/v) formic acid (FA) in water (MS buffer A) and 90% (v/v) ACN/0.1% (v/v) FA (MS buffer B). A gradient was run at 200 mL/min: 0–0.3 min, 5% B; 0.3–3 min, 5–100% B; 4–4.1 min, 100–5% B; 4.1–4.6 min, 5% B. Eluent was diverted to waste at 0–1 and 4–4.6 min acquisition time using an in-line divert valve. Multiple reaction monitoring in positive ion mode was used for compound detection.

### 4.17 Statistics and experimental design

All data in figures are presented as mean ± S.E.M., unless stated otherwise in the figure legend. Statistical analysis was performed using either one or two-way ANOVA with the suitable post-hoc correction for multiple comparisons as described in the figure legend. Where only two groups were compared, statistical significance was assessed by two-tailed Student's unpaired *t*-test. A *p* value of less than 0.05 was considered significant. Statistics were calculated in Prism 9.0 software (GraphPad Software Inc, USA).

## Author contributions

A.V.G, A.M.J., A.M.C. and M.P.M conceived and designed the studies. A.V.G. carried out cardiomyocyte experiments. A.M.C carried out live cell imaging experiments. H.A.P. carried out succinate measurements. G.R.B and N. B. carried out MitoB and CoQ analyses on the LC-MS/MS. N.B. developed and established the LC-MS/MS assay to measure CoQ. A.V.G, A.M.C, A.M.J and M.P.M. interpreted data. A.V.G. and M.P.M. wrote the original manuscript and all authors reviewed the revised manuscript.

## Declaration of competing interest

We have no conficts of interest to declare.

## Abbreviations

IR: ischemia reperfusion
AR: anoxia reoxygenation
ATP: adenosine triphosphate
ADP: adenosine diphosphate
MCT: monocarboxylate transporter
LDH: lactate dehydrogenase
NAD: nicotinamide adenine dinucleotide
NADH: dihydronicotinamide adenine dinucleotide
MPTP: mitochondrial permeability transition pore
CsA: cyclosporin A
Δψ_m_: mitochondrial membrane potential
Δψ_p_: plasma membrane potential
TMRM: tetramethyl-rhodamine methyl ester perchlorate
FCCP: carbonyl cyanide *p*-trifluoro-methoxyphenyl hydrazone
MtDNA: mitochondrial desoxyribonucleic acid
MDA: malondialdehyde
OXPHOS: oxidative phosphorylation
LC-MS/MS: liquid chromatography tandem mass spectrometry
PCR: polymerase chain reaction
TBARS: thiobarbituric acid reactive substances
