# Supplementary material for: Mitochondrial metabolism and bioenergetic function in an anoxic isolated adult mouse cardiomyocyte model of in vivo cardiac ischemia-reperfusion injury
Source: Redox Biol. 2022 Jun 17;54:102368. doi: 10.1016/j.redox.2022.102368 (PMC9234472; doi:10.1016/j.redox.2022.102368)
Supplement: Multimedia component 1 [file mmc1.docx]

**Supplementary Figure**

Mitochondrial metabolism and bioenergetic function in an anoxic isolated adult mouse cardiomyocyte model of *in vivo* cardiac ischemia-reperfusion injury

Anja V. Gruszczyk^a, b, c^, Alva M. Casey^a^, Andrew M. James^a^, Hiran A. Prag^a,d^, Nils Burger^a^, Georgina R. Bates^a^, Andrew R. Hall^a^, Fay M. Allen^a^, Thomas Krieg^d^, Kourosh Saeb-Parsy^b,c^, Michael P. Murphy^a,d,*^

^a^ *MRC Mitochondrial Biology Unit, Biomedical Campus, University of Cambridge, Cambridge CB2 0XY, UK*

^b^ *Department of Surgery and Cambridge NIHR Biomedical Research Centre, Biomedical Campus, University of Cambridge, Cambridge, CB2 2QQ, UK*

^c^ *NIHR Biomedical Research Centre and NIHR Blood and Transplant Research Unit in Organ Donation and Transplantation, Cambridge Biomedical Campus, Cambridge, UK*

^d^ *Department of Medicine, University of Cambridge, Cambridge, CB2 0QQ, UK*

^*^Corresponding author. MRC Mitochondrial Biology Unit, Biomedical Campus, University of Cambridge, Cambridge CB2 0XY, UK

*E-mail address:* [mpm@mrc-mbu.cam.ac.uk](mailto:mpm@mrc-mbu.cam.ac.uk)


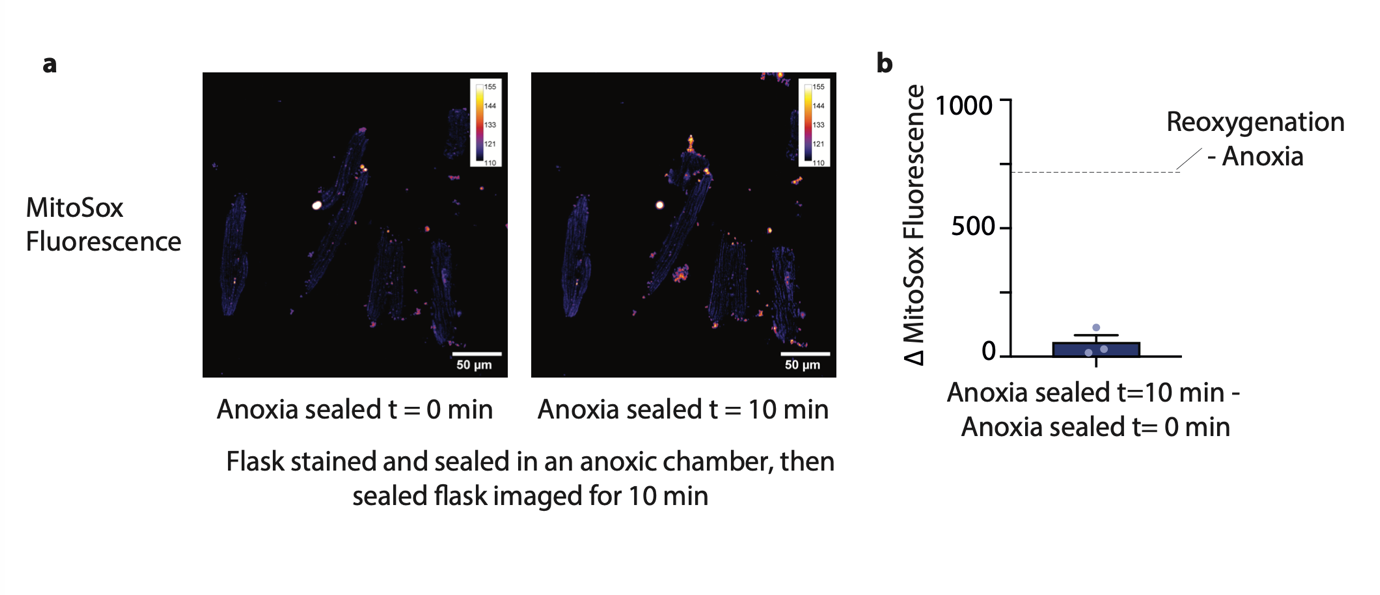


***Supplementary Figure 1****: Evaluation of duration of anoxia in sealed flasks*

(a) Cardiomyocytes were incubated for 30 minutes under anoxia. MitoSOX (10 µM) was added for the last 15 min of anoxia and then the flasks were sealed under anoxia. The sealed flasks were then transferred to room air and MitoSOX fluorescence in the sealed dish was imaged immediately, or after 10 min further incubation. There was no increase in MitoSOX fluorescence over this time. In contrast incubation under normoxia for 5 min led to extensive MitoSOX fluorescence (data shown in Figure 7 (d)), consistent with the cells remaining anoxic within the sealed flask over at least 10 min. n = 3 biological replicates.
